# SyReNN: A Tool for Analyzing Deep Neural Networks

**DOI:** 10.1007/978-3-030-72013-1_15

**Published:** 2021-02-26

**Authors:** Matthew Sotoudeh, Aditya V. Thakur

**Affiliations:** 8 Eindhoven University of Technology, Eindhoven, The Netherlands; 9 Aalborg University, Aalborg East, Denmark; University of California, Davis, CA 95616 USA

**Keywords:** Deep Neural Networks, Symbolic representation, Integrated Gradients

## Abstract

Deep Neural Networks (DNNs) are rapidly gaining popularity in a variety of important domains. Formally, DNNs are complicated vector-valued functions which come in a variety of sizes and applications. Unfortunately, modern DNNs have been shown to be vulnerable to a variety of attacks and buggy behavior. This has motivated recent work in formally analyzing the properties of such DNNs. This paper introduces SyReNN, a tool for understanding and analyzing a DNN by computing its *symbolic representation.* The key insight is to decompose the DNN into linear functions. Our tool is designed for analyses using *low-dimensional subsets* of the input space, a unique design point in the space of DNN analysis tools. We describe the tool and the underlying theory, then evaluate its use and performance on three case studies: computing Integrated Gradients, visualizing a DNN’s decision boundaries, and patching a DNN.

## 1 Introduction

Deep Neural Networks (DNNs) [18] have become the state-of-the-art in a variety of applications including image recognition [53,33] and natural language processing [12]. Moreover, they are increasingly used in safety- and security-critical applications such as autonomous vehicles [31] and medical diagnosis [10,38,28,37]. These advances have been accelerated by improved hardware and algorithms.

DNNs (Section 2) are programs that compute a vector-valued function, i.e., from $\mathbb{R}^n$ to $\mathbb{R}^m$. They are straight-line programs written as a concatenation of alternating linear and non-linear *layers*. The coefficients of the linear layers are learned from data via *gradient descent* during a training process. A number of different non-linear layers (called *activation functions*) are commonly used, including the *rectified linear* and *maximum pooling* functions.

Owing to the variety of application domains and deployment constraints, DNNs come in many different sizes. For instance, large image-recognition and

* Artifact available at https://zenodo.org/record/4124489. Extended paper available at https://arxiv.org/abs/2101.03263.

natural-language processing models are trained and deployed using cloud resources [33,12], medium-size models could be trained in the cloud but deployed on hardware with limited resources [31], and finally small models could be trained and deployed directly on edge devices [47,9,22,34,35]. There has also been a recent push to compress trained models to reduce their size [24]. Such smaller models play an especially important role in privacy-critical applications, such as wake word detection for voice assistants, because they allow sensitive user data to stay on the user's own device instead of needing to be sent to a remote computer for processing.

Although DNNs are very popular, they are not perfect. One particularly concerning development is that modern DNNs have been shown to be extremely vulnerable to *adversarial examples,* inputs which are intentionally manipulated to appear unmodified to humans but become misclassified by the DNN [54,19,40,8]. Similarly, *fooling examples* are inputs that look like random noise to humans, but are classified with high confidence by DNNs [41]. Mistakes made by DNNs have led to loss of life [36,17] and wrongful arrests [26,27]. For this reason, it is important to develop techniques for analyzing, understanding, and repairing DNNs.

This paper introduces SyReNN, a tool for understanding and analyzing DNNs. SyReNN implements state-of-the-art algorithms for computing precise symbolic representations of piecewise-linear DNNs (Section 3). Given an input subspace of a DNN, SyReNN computes a symbolic representation that decomposes the behavior of the DNN into finitely-many linear functions. SyReNN implements the one-dimensional analysis algorithm of Sotoudeh and Thakur [50] and extends it to the two-dimensional setting as described in Section 4.

***Key insights.*** There are two key insights enabling this approach, first identified in Sotoudeh and Thakur [50]. First, most popular DNN architectures today are *piecewise-linear*, meaning they can be precisely decomposed into finitely-many linear functions. This allows us to reduce their analysis to equivalent questions in linear algebra, one of the most well-understood fields of modern mathematics. Second, many applications only require analyzing the behavior of the DNN on a *low-dimensional subset* of the input space. Hence, whereas prior work has attempted to give up precision for efficiency in analyzing high-dimensional input regions [48,49,16], our work has focused on algorithms that are *both efficient and precise* in analyzing lower-dimensional regions (Section 4).

***Tool design.*** The SyReNN tool is designed to be easy to use and extend, as well as efficient (Section 5). The core of SyReNN is written as a highly-optimized, parallel C++ server using Intel TBB for parallelization [45] and Eigen for matrix operations [23]. A user-friendly Python front-end interfaces with the PyTorch deep learning framework [44].

***Use cases.*** We demonstrate the utility of SyReNN using three applications. The first computes *Integrated Gradients* (IG), a state-of-the-art measure used to determine which input dimensions (e.g., pixels for an image-recognition network) were most important in the final classification produced by the network (Section 6.1). The second precisely visualizes the decision boundaries of a DNN (Section 6.2). The last *patches* (repairs) a DNN to satisfy some desired specification involving infinitely-many points (Section 6.3). Thus, SyReNN is an interesting and useful tool in the toolbox for understanding and analyzing DNNs.

***Contributions.*** The contributions of this paper are:

- A definition of symbolic representation of DNNs (Section 3).
- An efficient algorithm for computing symbolic representations for DNNs over low-dimensional input subspaces (Section 4).
- A design of a usable and well-engineered tool implementing these ideas called SyReNN (Section 5).
- Three applications of SyReNN (Section 6).

Section 2 presents preliminaries about DNNs; Section 7 presents related work; Section 8 concludes. SyReNN is available on GitHub at https://github.com/95616ARG/SyReNN.

## 2 Preliminaries

We now formally define the notion of *DNN* we will use in this paper.

**Definition 1.** *A* Deep Neural Network *(DNN) is a function* $f : \mathbb{R}^n \to \mathbb{R}^m$ *which can be written* $f = f_1 \circ f_2 \cdots \circ f_n$ *for a sequence of* layer functions $f_1$, $f_2$, $\ldots$, $f_n$.

Our work is primarily concerned with the popular class of *piecewise-linear* DNNs, defined below. In this definition and the rest of this paper, we will use the term "polytope" to mean a *convex and bounded* polytope except where specified.

**Definition 2.** *A function* $f : \mathbb{R}^n \to \mathbb{R}^m$ *is* piecewise-linear (PWL) *if its input domain* $\mathbb{R}^n$ *can be partitioned into finitely-many possibly-unbounded polytopes* $X_1, X_2, \ldots, X_k$ *such that* $f_{\restriction X_i}$ *is* linear *for every* $X_i$.

The most common activation function used today is the ReLU function, a PWL activation function which is defined below.

**Definition 3.** *The* rectified linear function *(ReLU) is a function* $\textsc{ReLU} : \mathbb{R}^n \to \mathbb{R}^m$ *defined component-wise by*

$$\textsc{ReLU}(\vec{v})_i := \begin{cases} 0 & \textit{if } v_i < 0 \\ v_i & \textit{otherwise,} \end{cases}$$

*where* $\textsc{ReLU}(\vec{v})_i$ *is the ith component of the vector* $\textsc{ReLU}(\vec{v})$ *and* $v_i$ *is the ith component of the vector* $\vec{v}$.

In order to see that $\textsc{ReLU}$ is PWL, we must show that its input domain $\mathbb{R}^n$ can be partitioned such that, in each partition, $\textsc{ReLU}$ is linear. In this case, we can use the orthants of $\mathbb{R}^n$ as our partitioning: within each orthant, the signs of the components do not change hence $\textsc{ReLU}$ is the linear function that just zeros out the negative components.

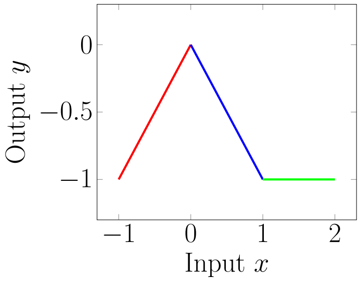

Fig. 1: Example function for which $\widehat{f_{\restriction[-1,2]}} = \{[-1,0],[0,1],[1,2]\}$.

Although we focus on ReLU due to its popularity and expository power, SyReNN works with a number of other popular PWL layers include MaxPool, Leaky ReLU, Hard Tanh, Fully-Connected, and Convolutional layers, as defined in [18]. PWL layers have become exceedingly common. In fact, nearly all of the state-of-the-art image recognition models bundled with Pytorch [43] are PWL.

*Example 1.* The DNN $f : \mathbb{R}^1 \to \mathbb{R}^1$ defined by

$$f(x) \coloneqq \begin{bmatrix} 1 & -1 & -1 \end{bmatrix} \text{ReLU}\left( \begin{bmatrix} 1 & -1 \\ 1 & 0 \\ -1 & 0 \end{bmatrix} \begin{bmatrix} x \\ 1 \end{bmatrix} \right)$$

can be broken into layers $f = f_1 \circ f_2 \circ f_3$ where

$$f_1(x) \coloneqq \begin{bmatrix} 1 & -1 \\ 1 & 0 \\ -1 & 0 \end{bmatrix} \begin{bmatrix} x \\ 1 \end{bmatrix}, \quad f_2 = \text{ReLU}, \quad \text{and} \quad f_3(\vec{v}) = \begin{bmatrix} 1 & -1 & -1 \end{bmatrix} \vec{v}.$$

The DNN's input-output behavior on the domain $[-1, 2]$ is shown in Figure 1.

## 3 A Symbolic Representation of DNNs

We formalize the symbolic representation according to the following definition:

**Definition 4.** *Given a PWL function $f : \mathbb{R}^n \to \mathbb{R}^m$ and a bounded convex polytope $X \subseteq \mathbb{R}^n$, we define the* symbolic representation of $f$ on $X$, *written $\widehat{f_{\restriction X}}$, to be a finite set of polytopes $\widehat{f_{\restriction X}} = \{P_1, \ldots, P_n\}$, such that:*

1. *The set $\{P_1, P_2, \ldots, P_n\}$ partitions $X$, except possibly for overlapping boundaries.*
2. *Each $P_i$ is a bounded convex polytope.*
3. *Within each $P_i$, the function $f_{\restriction P_i}$ is linear.*

Notably, if $f$ is a DNN using only PWL layers, then $f$ is PWL and so we can define $\widehat{f_{\restriction X}}$. This symbolic representation allows one to reduce questions about the DNN $f$ to questions about finitely-many linear functions $F_i$. For example, because linear functions are convex, to verify that $\forall x \in X.\quad f(x) \in Y$ for some polytope $Y$, it suffices to verify $\forall P_i \in \widehat{f_{\restriction X}}.\forall \vec{v} \in \mathtt{Vert}(P_i).\quad f(\vec{v}) \in Y$, where $\mathtt{Vert}(P_i)$ is the (finite) set of vertices for the bounded convex polytope $P_i$; thus, here both of the quantifiers are over finite sets. The symbolic representation described above can be seen as a generalization of the ExactLine representation [50], which considered only *one-dimensional* restriction domains of interest.

*Example 2.* Consider again the DNN $f : \mathbb{R}^1 \to \mathbb{R}^1$ given by

$$f(x) \coloneqq \begin{bmatrix} 1 & -1 & -1 \end{bmatrix} \mathrm{ReLU}\left( \begin{bmatrix} 1 & -1 \\ 1 & 0 \\ -1 & 0 \end{bmatrix} \begin{bmatrix} x \\ 1 \end{bmatrix} \right)$$

and the region of interest $X = [-1, 2]$. The input-output behavior of $f$ on $X$ is shown in Figure 1. From this, we can see that

$$\widehat{f_{\restriction X}} = \{[-1, 0], [0, 1], [1, 2]\}.$$

Within each of these partitions, the input-output behavior is *linear,* which for $\mathbb{R}^1 \to \mathbb{R}^1$ we can see visually as just a line segment. As this set fully partitions $X$, then, this is a valid $\widehat{f_{\restriction X}}$.

## 4 Computing the Symbolic Representation

This section presents an efficient algorithm for computing $\widehat{f_{\restriction X}}$ for a DNN $f$ composed of PWL layers. To retain both scalability and precision, we will *require the input region X be two-dimensional.* This design choice is relatively unexplored in the neural-network analysis literature (most analyses strike a balance between precision and scalability, ignoring dimensionality). We show that, for two-dimensional $X$, we can use an efficient polytope representation to produce an algorithm that demonstrates good best-case and in-practice efficiency while retaining full precision. This algorithm represents a direct generalization of the approach of [50].

The difficulties our algorithm addresses arise from three areas. First, when computing $\widehat{f_{\restriction X}}$ there may be exponentially many such partitions on all of $\mathbb{R}^n$ but only a small number of them may intersect with $X$. Consequently, the algorithm needs to be able to find those partitions that intersect with $X$ efficiently without explicitly listing all of the partitions on $\mathbb{R}^n$. Second, it is often more convenient to specify the partitioning via *hyperplanes separating the partitions* than explicit polytopes. For example, for the one-dimensional ReLU function we may simply state that the line $x = 0$ separates the two partitions, because ReLU is linear both in the region $x \leq 0$ and $x \geq 0$. Finally, neural networks are typically composed of sequences of linear and piecewise-linear layers, where the partitioning imposed by each layer individually may be well-understood but their composition is more complex. For example, identifying the linear partitions of $y = \textsc{ReLU}(4 \cdot \textsc{ReLU}(-3x - 1) + 2)$ is non-trivial, even though we know the linear partitions of each composed function individually.

Our algorithm only requires the user to specify the hyperplanes defining the partitioning for the activation function used in each layer; our current implementation comes with support for common PWL activation functions. For example, if a ReLU layer is used for an $n$-dimensional input vector, then the hyperplanes would be defined by the equations $x_1 = 0, x_2 = 0, \ldots, x_n = 0$. It then computes the symbolic representation for a *single layer at a time*, composing them sequentially to compute the symbolic representation across the entire network.

To allow such compositions of layers, instead of directly computing $\widehat{f_{\restriction X}}$, we will define another primitive, denoted by the operator $\otimes$ and sometimes referred to as Extend, such that

$$\textsc{Extend}(h, \widehat{g}) = h \otimes \widehat{g} = \widehat{h \circ g}. \tag{1}$$

Consider $f = f_n \circ f_{n-1} \circ \cdots \circ f_1$, and let $I : x \mapsto x$ be the identity map. $I$ is linear across its entire input space, and, thus, $\widehat{I_{\restriction X}} = \{X\}$. By the definition of $\textsc{Extend}(f_1, \cdot)$, we have $f_1 \otimes \widehat{I_{\restriction X}} = \widehat{(f_1 \circ I)_{\restriction X}} = \widehat{f_1{}_{\restriction X}}$, where the final equality holds by the definition of the identity map $I$. We can then iteratively apply this procedure to inductively compute $\widehat{(f_i \circ \cdots \circ f_1)_{\restriction X}}$ from $\widehat{(f_{i-1} \circ \cdots f_1)_{\restriction X}}$ like so:

$$f_i \otimes \widehat{(f_{i-1} \circ \cdots \circ f_1)_{\restriction X}} = \widehat{(f_i \circ f_{i-1} \circ \cdots \circ f_1)_{\restriction X}}$$

until we have computed $\widehat{(f_n \circ f_{n-1} \circ \cdots \circ f_1)_{\restriction X}} = \widehat{f_{\restriction X}}$, which is the required symbolic representation.

### 4.1 Algorithm for Extend

Algorithm 1 present an algorithm for computing Extend for arbitrary PWL functions, where $\textsc{Extend}(h, \widehat{g}) = h \otimes \widehat{g} = \widehat{h \circ g}$.

***Geometric intuition for the algorithm.*** Consider the ReLU function (Definition 3). It can be shown that, within any orthant (i.e., when the signs of all coefficients are held constant), $\textsc{ReLU}(\vec{x})$ is equivalent to some linear function, in particular the element-wise product of $\vec{x}$ with a vector that zeroes out the negative-signed components. However, for our algorithm, all we need to know is that the linear partitions of ReLU (in this case the orthants) are separated by hyperplanes $x_1 = 0, x_2 = 0, \ldots, x_n = 0$.

Given a two-dimensional convex bounded polytope $X$, the execution of the algorithm for $f = \textsc{ReLU}$ can be visualized as follows. We pick some vertex $v$ of $X$, and begin traversing the boundary of the polytope in counter-clockwise order. If we hit an orthant boundary (corresponding to some hyperplane $x_i = 0$), it implies that the behavior of the function behaves differently at the points of the polytope to one side of the boundary from those at the other side of the boundary. Thus, we *partition $X$ into $X_1$ and $X_2$*, where $X_1$ lies to one side of the hyperplane and $X_2$ lies to the other side. We recursively apply this procedure to $X_1$ and $X_2$ until the resulting polytopes all lie on exactly one side of every hyperplane (orthant boundary). But lying on exactly one side of every hyperplane (orthant boundary) implies each polytope lies entirely within a linear partition of the function (a single orthant), hence the application of the function on that polytope is linear, and hence we have our partitioning.

***Functions used in algorithm.*** Given a two-dimensional bounded convex polytope $X$, `Vert(`$X$`)` returns a list of its vertices in counter-clockwise order, repeating the initial vertex at the end. Given a set of points $X$, `ConvexHull(`$X$`)` represents their convex hull (the smallest bounded polytope containing every point in $X$). Given a scalar value $x$, `Sign(`$x$`)` computes the sign of that value (i.e., $-1$ if $x < 0$, $+1$ if $x > 0$, and 0 if $x = 0$).

***Algorithm description.*** The key insight of the algorithm is to recursively partition the polytopes until such a partition lies entirely within a linear region of the function $f$. Algorithm 1 begins by constructing a queue containing the polytopes of $\widehat{g_{\restriction X}}$. Each iteration either removes a polytope from the queue that lies entirely in one linear region (placing it in $Y$), or splits (partitions) some polytope into two smaller polytopes that get put back into the queue. When we pop a polytope $P$ from the queue, Line 6 iterates over all hyperplanes $N_k \cdot x = b_k$ defining the piecewise-linear partitioning of $f$, looking for any for which some vertex $V_i$ lies on the positive side of the hyperplane and another vertex $V_j$ lies on the negative side of the hyperplane. If none exist (Line 7), by convexity we are guaranteed that the entire polytope lies entirely on one side with respect to every hyperplane, meaning it lies entirely within a linear partition of $f$. Thus, we can add it to $Y$ and continue. If two such vertices are found (starting Line 10), then we can find "extreme" $i$ and $j$ indices such that $V_i$ is the last vertex in a counter-clockwise traversal to lie on the same side of the hyperplane as $V_1$ and $V_j$ is the last vertex lying on the opposite side of the hyperplane. We then call `SplitPlane()` (Algorithm 2) to actually partition the polytope on opposite sides of the hyperplane, adding both to our worklist.

In the best case, each partition is in a single orthant: the algorithm never calls `SplitPlane()` at all — it merely iterates over all of the $n$ input partitions, checks their $v$ vertices, and appends to the resulting set (for a best-case complexity of $O(nv)$). In the worst case, it splits each polytope in the queue on each face, resulting in exponential time complexity. As we will show in Section 6, this exponential worst-case behavior is not encountered in practice, thus making SyReNN a practical tool for DNN analysis.

Please see the extended version of this paper for a worked example of the algorithm's execution.

### 4.2 Representing Polytopes

We close this section with a discussion of implementation concerns when representing the convex polytopes that make up the partitioning of $\widehat{f_{\restriction X}}$. In standard computational geometry, bounded polytopes can be represented in two equivalent forms:

**Algorithm 1:** $f \otimes \widehat{g_{\restriction X}}$ for two-dimensional $X$. $f$ is defined by hyperplanes $N_1 \cdot x = b_1$ through $N_m \cdot x = b_m$ such that, within any partition imposed by the hyperplanes $f$ is equivalent to some affine function.

**Input:** $\widehat{g_{\restriction X}} = \{P_1, \ldots, P_n\}$.
**Output:** $\widehat{f \circ g_{\restriction X}}$

$W \leftarrow$ `ConstructQueue`$(\widehat{g_{\restriction X}})$
$Y \leftarrow \emptyset$
**while** $W$ *not empty* **do**
    $P \leftarrow$ `Pop`$(W)$
    $V \leftarrow$ `Vert`$(P)$
    $K \leftarrow \{N_k \mid \exists i, j : \texttt{Sign}(N_k \cdot g(V_i) - b_k) > 0 \wedge \texttt{Sign}(N_k \cdot g(V_j) - b_k) < 0\}$
    **if** $K = \emptyset$ **then**
        $Y \leftarrow Y \cup \{P\}$
        **continue**
    $N, b \leftarrow$ any element from $K$
    $i \leftarrow \arg\max_i \{\texttt{Sign}(N \cdot g(V_i) - b) = \texttt{Sign}(N \cdot g(V_1) - b)\}$
    $j \leftarrow \arg\max_j \{\texttt{Sign}(N \cdot g(V_j) - b) \neq \texttt{Sign}(N \cdot g(V_i) - b)\}$
    **for** $V' \in \texttt{SplitPlane}(V, g, i, j, N, b)$ **do**
        $W \leftarrow$ `Push`$(W,$ `ConvexHull`$(V'))$
**return** $Y$

1. The *half-space* or *H-representation*, which encodes the polytope as an intersection of finitely-many half-spaces. (Each half-space being defined as a halfspace defined by an affine inequality $Ax \leq b$.)
2. The *vertex* or *V-representation*, which encodes the polytope as a set of finitely many points; the polytope is then taken to be the convex hull of the points (i.e., smallest convex shape containing all of the points).

Certain operations are more efficient when using one representation compared to the other. For example, finding the intersection of two polytopes in an H-representation can be done in linear time by concatenating their representative half-spaces, but the same is not possible in V-representation.

There are two main operations on polytopes we need perform in our algorithms: (i) splitting a polytope with a hyperplane, and (ii) applying an affine map to all points in the polytope. In general, the first is more efficient in an H-representation, while the latter is more efficient in a V-representation. However, when restricted to two-dimensional polygons, the former is also efficient in a V-representation, as demonstrated by Algorithm 2, helping to motivate our use of the V-representation in our algorithm.

Furthermore, the two polytope representations have different resiliency to floating-point operations. In particular, H-representations for polytopes in $\mathbb{R}^n$ are notoriously difficult to achieve high-precision with, because the error introduced from using floating point numbers gets arbitrarily large as one goes in a particular direction along any hyperplane face. Ideally, we would like the

**Algorithm 2:** SplitPlane$(V, g, i, j, N, b)$

**Input:** $V$, the vertices of the polytope in the input space of $g$. A function $g$. $i$ is the index of the last vertex lying on the same side of the orthant face as $V_1$. $j$ is the index of the last vertex lying on the opposite side of the orthant face as $V_1$. $N$ and $b$ define the hyperplane $N \cdot x = b$ to split on.

**Output:** $\{P_1, P_2\}$, two sets of vertices whose convex hulls form a partitioning of $V$ such that each lies on only one side of the $N \cdot x = b$ hyperplane.

$p_i \leftarrow V_i + \frac{b - N \cdot g(V_i)}{N \cdot (g(V_{i+1}) - g(V_i))}(V_{i+1} - V_i)$

$p_j \leftarrow V_j + \frac{b - N \cdot g(V_j)}{N \cdot (g(V_{j+1}) - g(V_j))}(V_{j+1} - V_j)$

$A \leftarrow \{p_i, p_j\} \cup \{v \in V \mid \texttt{Sign}(N \cdot v - b) = \texttt{Sign}(N \cdot V_i - b)\}$

$B \leftarrow \{p_i, p_j\} \cup \{v \in V \mid \texttt{Sign}(N \cdot v - b) = \texttt{Sign}(N \cdot V_j - b)\}$

**return** $\{A, B\}$

hyperplane to be most accurate in the region of the polytope itself, which corresponds to choosing the magnitude of the norm vector correctly. Unfortunately, to our knowledge, there is no efficient algorithm for computing the ideal floating point H-representation of a polytope, although libraries such as APRON [30] are able to provide reasonable results for low-dimensional spaces. However, because neural networks utilize extremely high-dimensional spaces (often hundreds or thousands of dimensions) and we wish to iteratively apply our analysis, we find that errors from using floating-point H-representations can quickly multiply and compound to become infeasible. By contrast, floating-point inaccuracies in a V-representation are directly interpretable as slightly misplacing the vertices of the polytope; no "localization" process is necessary to penalize inaccuracies close to the polytope more than those far away from it.

Another difference is in the space complexity of the representation. In general, H-representations can be more space-efficient for common shapes than V-representations. However, when the polytope lies in a low-dimensional subspace of a larger space, the V-representation is usually significantly more efficient.

Thus, V-representations are a good choice for low-dimensionality polytopes embedded in high-dimensional space, which is exactly what we need for analyzing neural networks with two-dimensional restriction domains of interest. This is why we designed our algorithms to rely on $\texttt{Vert}(X)$, so that they could be directly computed on a V-representation.

The 2D algorithm described above can be seen as implementing the recursive case of a more general, $n$-dimensional version of the algorithm that recurses on each of the $(n - 1)$-dimensional facets. Please see the extended version of this paper for more details.

## 5 SyReNN tool

This section provides more details about the design and implementation of our tool, SyReNN (Symbolic Representations of Neural Networks), which computes

$\widehat{f_{\restriction X}}$, where $f$ is a DNN using only piecewise-linear layers and $X$ is a union of one- or two-dimensional polytopes. The tool is available under the MIT license at https://github.com/95616ARG/SyReNN and in the PyPI package `pysyrenn`.

***Input and output format.*** SyReNN supports reading DNNs from two standard formats: ERAN (a textual format used by the ERAN project [1]) as well as ONNX (an industry-standard format supporting a wide variety of different models) [42]. Internally, the input DNN is described as an instance of the `Network` class, which is itself a list of sequential `Layers`. A number of layer types are provided by SyReNN, including `FullyConnectedLayer`, `ConvolutionalLayer`, and `ReLULayer`. To support more complicated DNN architectures, we have implemented a `ConcatLayer`, which represents a concatenation of the output of two different layers. The input region of interest, $X$, is defined as a polytope described by a list of its vertices in counter-clockwise order. The output of the tool is the symbolic representation $\widehat{f_{\restriction X}}$.

***Overall Architecture.*** We designed SyReNN in a client-server architecture using gRPC [20] and protocol buffers [21] as a standard method of communication between the two. This architecture allows the bulk of the heavy computation to be done in efficient C++ code, while allowing user-friendly interfaces in a variety of languages. It also allows practitioners to run the server remotely on a more powerful machine if necessary. The C++ server implementation uses the Intel TBB library for parallelization. Our official front-end library is written in Python, and available as a package on PyPI so installation is as simple as `pip install pysyrenn`. The entire project can be built using the Bazel build system, which manages dependencies using checksums.

***Server Architecture.*** The major algorithms are implemented as a gRPC server written in C++. When a connection is first made, the server initializes the state with an empty DNN $f(x) = x$. During the session, three operations are permitted: (i) append a layer $g$ so that the current session's DNN is updated from $f_0$ to $f_1(x) \coloneqq g(f_0(x))$, (ii) compute $\widehat{f_{\restriction X}}$ for a one-dimensional $X$, or (iii) compute $\widehat{f_{\restriction X}}$ for a two-dimensional $X$. We have separate methods for one- and two-dimensional $X$, because the one-dimensional case has specific optimizations for controlling memory usage. The `SegmentedLine` and `UPolytope` types are used to represent one- and two-dimensional partitions of $X$, respectively. When operation (1) is performed, a new instance of the `LayerTransformer` class is initialized with the relevant parameters and added to a running `vector` of the current layers. When operation (2) is performed, a new queue of `SegmentedLines` is constructed, corresponding to $X$, and the before-allocated `LayerTransformers` are applied sequentially to compute $\widehat{f_{\restriction X}}$. In this case, extra control is provided to automatically gauge memory usage and pause computation for portions of $X$ until more memory is made available. Finally, when operation (3) is a performed, a new instance of `UPolytope` is initialized with the vertices of $X$ and the `LayerTransformers` are again applied sequentially to compute $\widehat{f_{\restriction X}}$.

***Client Architecture.*** Our Python client exposes an interface for defining DNNs similar to the popular Sequential-Network Keras API [11]. Objects represent individual layers in the network, and they can be combined sequentially into a `Network` instance. The key addition of our library is that this `Network` exposes methods for computing $\widehat{f_{\restriction X}}$ given a V-representation description of $X$. To do this, it invokes the server and passes a layer-by-layer description of $f$ followed by the polytope $X$, then parses the response $\widehat{f_{\restriction X}}$.

***Extending to support different layer types.*** Different layer types are supported by sub-classing the `LayerTransformer` class. Instances of this class expose a method for computing $\textsc{Extend}(h, \cdot)$ for the corresponding layer $h$. To simplify implementation, two sub-classes of `LayerTransformer` are provided: one for entirely-linear layers (such as fully-connected and convolutional layers), and one for piecewise-linear layers. For fully-linear layers, all that needs to be provided is a method computing the layer function itself. For piecewise-linear layers, two methods need to be provided: (1) computing the layer function itself, and (2) one describing the hyperplanes which separate the linear regions. The base class then directly implements Algorithm 1 for that layer. This architecture makes supporting new layers a straight-forward process.

***Float Safety.*** Like Reluplex [32], SyReNN uses floating-point arithmetic to compute $\widehat{f_{\restriction X}}$ efficiently. Unfortunately, this means that in some cases its results will not be entirely precise when compared to a real-valued or multiple-precision version of the algorithm. Approaches for addressing this are discussed in the extended version of this paper.

## 6 Applications of SyReNN

This section presents the use of SyReNN in three example case studies.

### 6.1 Integrated Gradients

A common problem in the field of *explainable machine learning* is understanding *why* a DNN made the prediction it did. For example, given an image classified by a DNN as a 'cat,' why did the DNN decide it was a cat instead of, say, a dog? Were there particular pixels which were particularly important in deciding this? Integrated Gradients (IG) [52] is the state-of-the-art method for computing such *model attributions.*

**Definition 5.** *Given a DNN $f$, the* integrated gradients along dimension $i$ for input $x$ and baseline $x'$ *is defined to be:*

$$IG_i(x) \overset{def}{=} (x_i - x'_i) \times \int_{\alpha=0}^{1} \frac{\partial f(x' + \alpha \times (x - x'))}{\partial x_i} d\alpha. \tag{2}$$

The computed value $IG_i(x)$ determines relatively how important the $i$th input (e.g., pixel) was to the classification.

However, exactly computing this integral requires a symbolic, closed form for the gradient of the network. Until [50], it was not known how to compute such a closed-form and so IGs were always only *approximated* using a sampling-based approach. Unfortunately, because it was unknown how to compute the true value, there was no way for practitioners to determine how accurate their approximations were. This is particularly concerning in fairness applications where an accurate attribution is exceedingly important.

In [50], it was recognized that, when $X = \texttt{ConvexHull}(\{x, x'\})$, $\widehat{f_{\restriction X}}$ can be used to *exactly* compute $IG_i(x)$. This is because within each partition of $\widehat{f_{\restriction X}}$ the gradient of the network is *constant* because it behaves as a linear function, and hence the integral can be written as the weighted sum of such finitely-many gradients.[1] Using our symbolic representation, the exact IG can thus be computed as follows:

$$\sum_{\texttt{ConvexHull}(\{y_i, y_i'\}) \in \widehat{f_{\restriction \texttt{ConvexHull}(\{x, x'\})}}} (y_i' - y_i) \times \frac{\partial f(0.5 \times (y_i + y_i'))}{\partial x_i} \tag{3}$$

Where here $y_i, y_i'$ are the endpoints of the segment with $y_i$ closer to $x$ and $y_i'$ closest to $x'$.

***Implementation.*** The helper class `IntegratedGradientsHelper` is provided by our Python client library. It takes as input a DNN $f$ and a set of $(x, x')$ input-baseline pairs and then computes IG for each pair.

***Empirical Results.*** In [50] SyReNN was used to show conclusively that existing sampling-based methods were insufficient to adequately approximate the true IG. This realization led to changes in the official IG implementation to use the more-precise trapezoidal sampling method we argued for.

***Timing Numbers.*** In those experiments, we used SyReNN to compute $\widehat{f_{\restriction X}}$ for three different DNNs $f$, namely the small, medium, and large convolutional models from [1]. For each DNN, we ran SyReNN on 100 one-dimensional lines. The 100 calls to SyReNN completed in 20.8 seconds for the small model, 183.3 for the medium model, and 615.5 for the big model. Tests were performed on an Intel Core i7-7820X CPU at 3.60GHz with 32GB of memory.

### 6.2 Visualization of DNN Decision Boundaries

Whereas IG helps understand why a DNN made a particular prediction about a single input point, another major task is *visualizing* the decision boundaries of a DNN on *infinitely-many* input points. Figure 2 shows a visualization of an ACAS Xu DNN [31] which takes as input the position of an airplane and an approaching attacker, then produces as output one of five advisories instructing the plane, such as "clear of conflict" or to move "weak left." Every point in the diagram represents the relative position of the approaching plane, while the color indicates the advisory.

[1] As noted in [50], this technically requires a slight strengthening of the definition of $\widehat{f_{\restriction X}}$ which is satisfied by our algorithms as defined above.

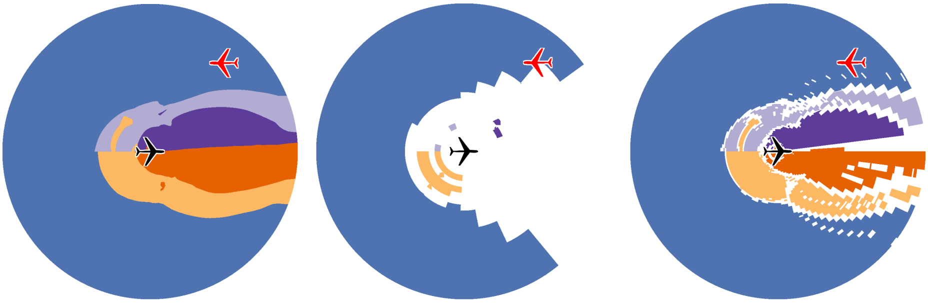

(a) Decision boundaries computed using $\widehat{f_{\restriction X}}$

(b) Decision boundaries computed using DeepPoly$[k = 25^2]$

(c) Decision boundaries computed using DeepPoly$[k = 100^2]$

Legend: Clear-of-Conflict, Weak Right, Strong Right, Strong Left, Weak Left.

Fig. 2: Visualization of decision boundaries for the ACAS Xu network. Using SyReNN (left) quickly produces the exact decision boundaries. Using abstract interpretation-based tools like DeepPoly (middle and right) are slower and produce only imprecise approximations of the decision boundaries.

One approach to such visualizations is to simply sample finitely-many points and extrapolate the behavior on the entire domain from those finitely-many points. However, this approach is imprecise and risks missing vital information because there is no way to know the correct sampling density to use to identify all important features.

Another approach is to use a tool such as DeepPoly [49] to over-approximate the output range of the DNN. However, because DeepPoly is a relatively coarse over-approximation, there may be regions of the input space for which it cannot state with confidence the decision made by the network. In fact, the approximations used by DeepPoly are extremely coarse. A naïve application of DeepPoly to this problem results in it being unable to make claims about *any* of the input space of interest. In order to utilize it, we must *partition* the space and run DeepPoly within each partition, which significantly slows down the analysis. Even when using $25^2$ partitions, Figure 2b shows that most of the interesting region is still unclassifiable with DeepPoly (shown in white). Only with $100^2$ partitions can DeepPoly effectively approximate the decision boundaries, although it is still quite imprecise.

By contrast, $\widehat{f_{\restriction X}}$ can be used to *exactly* determine the decision boundaries on any 2D polytope subset of the input space, which can then be plotted. This is shown in Figure 2a. Furthermore, as shown in Table 1, the approach using $\widehat{f_{\restriction X}}$ is *significantly* faster than that using ERAN, even as we get the *precise* answer instead of an approximation. Such visualizations can be particularly helpful in identifying issues to be fixed using techniques such as those in Section 6.3.

Table 1: Comparing the performance of DNN visualization using SyReNN versus DeepPoly for the ACAS Xu network [31]. $\widehat{f_{\restriction X}}$ size is the number of partitions in the symbolic representation. SyReNN time is the time taken to compute $\widehat{f_{\restriction X}}$ using SyReNN. DeepPoly[$k$] time is the time taken to compute DeepPoly for approximating decision boundaries with $k$ partitions. Each scenario represents a different two-dimensional slice of the input space; within each slice, the heading of the intruder relative to the ownship along with the speed of each involved plane is fixed.

| | | | DeepPoly time (secs) | | |
|---|---|---|---|---|---|
| Scenario | $\widehat{f_{\restriction X}}$ size | SyReNN time (secs) | k = $25^2$ | k = $55^2$ | k = $100^2$ |
| Head-On, Slow | 33200 | 10.9 | 9.1 | 43.2 | 141.3 |
| Head-On, Fast | 30769 | 10.2 | 8.2 | 39.0 | 128.0 |
| Perpendicular, Slow | 37251 | 12.5 | 9.2 | 42.9 | 141.7 |
| Perpendicular, Fast | 33931 | 11.4 | 8.2 | 39.2 | 127.5 |
| Opposite, Slow | 36743 | 12.1 | 9.8 | 46.7 | 152.5 |
| Opposite, Fast | 38965 | 13.0 | 9.5 | 45.2 | 147.3 |
| -Perpendicular, Slow | 36037 | 11.9 | 9.5 | 45.0 | 146.4 |
| -Perpendicular, Fast | 33208 | 10.9 | 8.3 | 39.5 | 130.2 |

***Implementation.*** The helper class `PlanesClassifier` is provided by our Python client library. It takes as input a DNN $f$ and an input region $X$, then computes the decision boundaries of $f$ on $X$.

***Timing Numbers.*** Timing comparisons are given in Table 1. We see that SyReNN is quite performant, and the exact SyReNN can be computed more quickly than even a mediocre approximation from DeepPoly using $55^2$ partitions. Tests were performed on a dedicated Amazon EC2 c5.metal instance, using BenchExec [5] to limit the number of CPU cores to 16 and RAM to 16GB.

### 6.3 Patching of DNNs

We have now seen how SyReNN can be used to visualize the behavior of a DNN. This can be particularly useful for identifying buggy behavior. For example, in Figure 2a we can see that the decision boundary between "strong right" and "strong left" is not symmetrical.

The final application we consider for SyReNN is *patching DNNs* to correct undesired behavior. Patching is described formally in [51]. Given an initial network $N$ and a *specification* $\phi$ describing desired constraints on the input/output, the goal of patching is to find a small modification to the parameters of $N$ producing a new DNN $N'$ that satisfies the constraints in $\phi$.

The key theory behind DNN patching we will use was developed in [51]. The key realization of that work is that, for a certain DNN architecture, correcting the network behavior on an infinite, 2D region $X$ is exactly equivalent to correcting

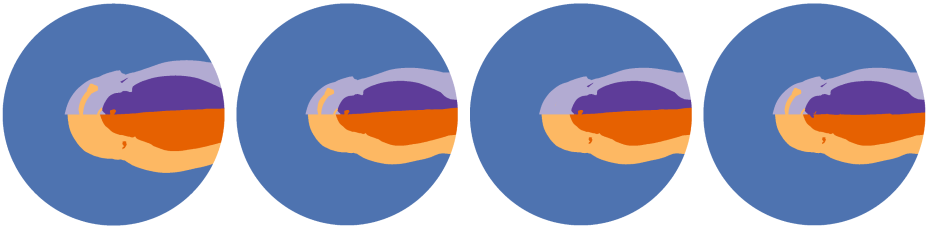

(a) Before patching. (b) Patched pockets. (c) Patched bands. (d) Patched symmetry.

Legend: Clear-of-Conflict, Weak Right, Strong Right, Strong Left, Weak Left.

Fig. 3: Network patching.

its behavior on *the finitely-many vertices* $\texttt{Vert}(P_i)$ for each of the finitely-many $P_i \in \widehat{f_{\restriction X}}$. Hence, SyReNN plays a key role in enabling efficient DNN patching.

For this case study, we patched the same aircraft collision-avoidance DNN visualized in Section 6.2. We patched the DNN three times to correct three different buggy behaviors of the network: (i) remove "Pockets" of strong left/strong right in regions that are otherwise weak left/weak right; (ii) remove the "Bands" of weak-left advisory behind and to the left of the plane; and (iii) enforce "Symmetry" across the horizontal. The DNNs before and after patching with different specifications are shown in Figure 3.

***Implementation*** The helper class `NetPatcher` is provided by our Python client library. It takes as input a DNN $f$ and pairs of input region, output label $X_i, Y_i$, then computes a new DNN $f'$ which maps all points in each $X_i$ into $Y_i$.

***Timing Numbers.*** As in Section 6.2, computing $\widehat{f_{\restriction X}}$ for use in patching took approximately 10 seconds.

## 7 Related Work

The related problem of exact reach set analysis for DNNs was investigated in [58]. However, the authors use an algorithm that relies on explicitly enumerating all exponentially-many ($2^n$) possible signs at each ReLU layer. By contrast, our algorithm adapts to the actual input polytopes, efficiently restricting its consideration to activations that are actually possible.

Hanin and Rolnick [25] prove theoretical properties about the cardinality of $\widehat{f_{\restriction X}}$ for ReLU networks, showing that $|\widehat{f_{\restriction X}}|$ is expected to grow polynomially with the number of nodes in the network for randomly-initialized networks.

Thrun [55] and Bastani et al.[4] extract symbolic rules meant to approximate DNNs, which can approximate the symbolic representation $\widehat{f_{\restriction X}}$.

In particular, the ERAN [1] tool and underlying DeepPoly [49] domain were designed to verify the non-existence of adversarial examples. Breutel et al. [6] give an iterative refinement algorithm for an overapproximation of the weakest precondition as a polytope where the required output is also a polytope.

Scheibler et al. [46] verify the safety of a machine-learning controller using the SMT-solver iSAT3, but support small unrolling depths and basic safety properties. Zhu et al. [60] use a synthesis procedure to generate a safe deterministic program that can enforce safety conditions by monitoring the deployed DNN and preventing potentially unsafe actions. The presence of adversarial and fooling inputs for DNNs as well as applications of DNNs in safety-critical systems has led to efforts to verify and certify DNNs [3,32,14,29,16,7,57,49,2]. *Approximate reachability analysis* for neural networks safely overapproximates the set of possible outputs [16,58,59,57,13,56].

Prior work in the area of network patching focuses on enforcing constraints on the network during training. DiffAI [39] is an approach to train neural networks that are certifiably robust to adversarial perturbations. DL2 [15] allows for training and querying neural networks with logical constraints.

## 8 Conclusion and Future Work

We presented SyReNN, a tool for understanding and analyzing DNNs. Given a piecewise-linear network and a low-dimensional polytope subspace of the input subspace, SyReNN computes a symbolic representation that decomposes the behavior of the DNN into finitely-many linear functions. We showed how to efficiently compute this representation, and presented the design of the corresponding tool. We illustrated the utility of SyReNN on three applications: computing exact IG, visualizing the behavior of DNNs, and patching (repairing) DNNs.

In contrast to prior work, SyReNN explores a unique point in the design space of DNN analysis tools. Instead of trading off precision of the analysis for efficiency, SyReNN focuses on analyzing DNN behavior on *low-dimensional subspaces* of the domain, for which we can provide *both* efficiency and precision.

We plan on extending SyReNN to make use of GPUs and other massively-parallel hardware to more quickly compute $\widehat{f_{\restriction X}}$ for large $f$ or $X$. Techniques to support input polytopes that are greater than two dimensional is also a ripe area of future work. We may also be able to take advantage of the fact that non-convex polytopes can be represented efficiently in 2D. Extending algorithms for $\widehat{f_{\restriction X}}$ to handle architectures such as Recurrent Neural Networks (RNNs) will open up new application areas for SyReNN.

*Acknowledgements.* We thank the anonymous reviewers for their feedback and suggestions on this work. This material is based upon work supported by a Facebook Probability and Programming award.
